# Effects of different cluster-set rest intervals during plyometric-jump training on measures of physical fitness: A randomized trial

**DOI:** 10.1371/journal.pone.0285062

**Published:** 2023-10-04

**Authors:** Behzad Taaty Moghadam, Hossein Shirvani, Rodrigo Ramirez-Campillo, Eduardo Báez-San Martín, Seyed Mojtaba Paydar Ardakani, Ali Abdolmohamadi, Behzad Bazgir

**Affiliations:** 1 Exercise Physiology Research Center, Lifestyle Institute, Baqiyatallah University of Medical Sciences, Tehran, Iran; 2 Exercise and Rehabilitation Sciences Institute, School of Physical Therapy, Faculty of Rehabilitation Sciences, Universidad Andres Bello, Santiago, Chile; 3 Sports Coach Career, School of Education, Universidad Viña del Mar, Viña del Mar, Chile; 4 Department of Sport Sciences, Faculty of Physical Activity and Sport Sciences, Universidad de Playa Ancha, Valparaíso, Chile; 5 Department of Sport Sciences, Ardakan University, Ardakan, Iran; 6 University of non-governmental Qadir, Langarood, Iran; University of Montenegro, MONTENEGRO

## Abstract

The optimal intra-set rest for cluster sets (CLS) during plyometric-jump training (PJT) to improve physical fitness remains unclear. The objective of this quasi-experimental study was to compare the effects of PJT with traditional (TRS) vs. CLS structures, using different intra-set rests, on the physical fitness of healthy participants. Forty-seven recreationally active young men performed 3–5 sets of 10–12 repetitions of upper- and lower-body PJT exercises twice a week for six weeks using different set configurations: TRS group (no intra-set rest), and the CLS10, CLS20 and CLS30 groups with 10, 20 and 30 s of intra-set rest, respectively, while the total rest period was equated. Pretest-posttest measurements were carried out 48 h before and after the intervention and the rating of fatigue (ROF) was also assessed using a numerical scale (0–10 points) 20 min after the first and last (i.e., 12^th^) session. There was no significant difference in the mean energy intake between groups (*p* > 0.05). The repeated measures ANOVA revealed that all groups showed similar improvements (*p* < 0.05) in body mass, body mass index, fat-free mass, one repetition maximum (dynamic strength) and repetitions to failure (muscular endurance) in back squat and chest press, handgrip strength, standing long jump, 20 m sprint, 9-m shuttle run (change of direction speed), and ROF. Of note, the ROF was lower for the CLS20 and CLS30 groups, independent from the training effect. The physical fitness of recreationally active young men improved after 6 weeks of PJT involving intra-set rest intervals of 0 s, 10 s, 20 s, or 30 s. However, an intra-set rest of 20 s and 30 s seems to induce lower exercise-induced fatigue perception.

## Introduction

Plyometric-jump training (PJT) is a popular training method [1]. PJT mainly includes jump exercises involving the stretch-shortening cycle of the muscle-tendon complex, that allow muscles to store energy during the deceleration phase and release it during the acceleration phase [2–4]. PJT can induce adaptations (e.g., increased motor unit recruitment; increased muscle fiber contraction velocity, strength and power; improved mechanical properties of the muscle–tendon complex) [5, 6] that may aid to improve muscle strength [6–10], jumping and sprinting performance [6, 8–12], change of direction speed (CODS) [11, 13–15], and anthropometrics [16–18].

However, optimal prescription of PJT variables is unclear at present [19], particularly for the recovery time between jump efforts. For example, among 27 analyzed items related to PJT prescription variables, inter-repetition rest time showed the highest (~75%) index of underreported information [19]. It has been suggested that 15 s between jump efforts may afford adequate recovery [20], although the cross-sectional nature of the findings precludes long-term extrapolation. Asadi and Ramírez-Campillo (2016) compared the effects of 6-week of PJT using different recovery strategies between jump efforts, including cluster sets (CLS) compared to traditional sets (TRS). The CLS group performed five sets of 20 (2 × 10) repetitions with a 30/90-s rest configuration, while the TRS group completed five sets of 20 repetitions with 120 s of rest between sets. The participants (male college students) from both groups improved physical fitness performance, although the CLS group achieved greater magnitude of improvement in vertical and horizontal jump performance, as well as in non-reactive agility (i.e., T test) [21].

The CLS incorporates a short rest period within a set of PJT, while maintaining rest periods between sets, with the aim to optimize the repetition performance and minimize the cumulative fatigue that is typically observed during TRS [22]. However, the optimal CLS configuration is unclear at present, with some studies using effective CLS configurations of 10 s [23] or 30 s [21], but without a comparison between CLS configurations. Therefore, the purpose of this study was to compare the effects of six weeks of PJT using CLS configurations with 10 s, 20 s and 30 s on the physical fitness of recreationally active men. We hypothesized that longer versus shorter intra-set rest periods would induce similar improvements in physical fitness and anthropometric factors, while inducing less muscle fatigue.

## Materials and methods

### Participants

**Fig 1:**
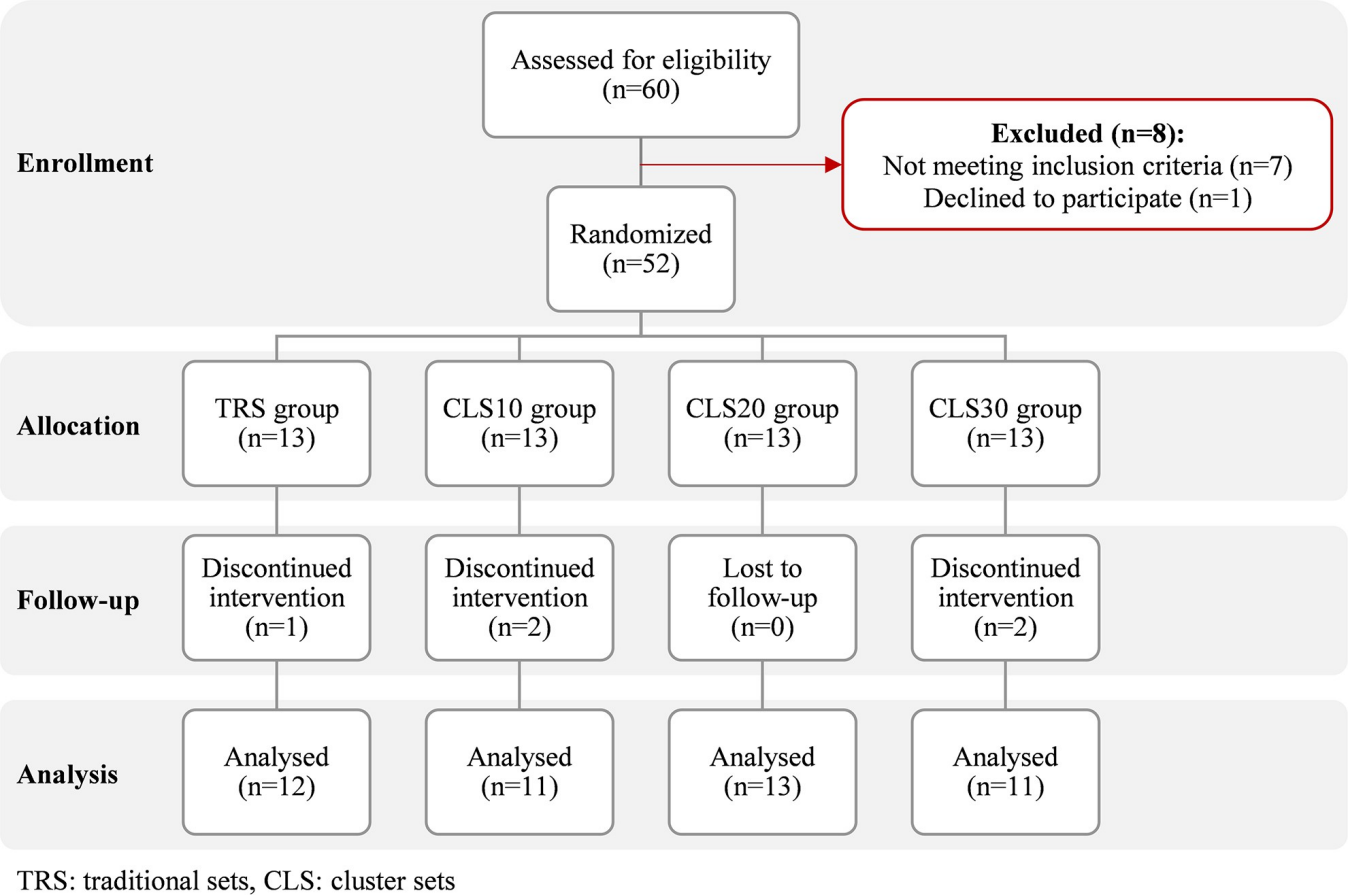
Recruitment flowchart from enrolment to data analysis.

This study was supposed to be conducted on military forces, but due to the lack of access to a sufficient number of the relevant participants, the population was changed to active young men. However, no other changes were made in the inclusion and exclusion criteria for selecting the subjects. Therefore, a total of 52 recreationally active men aged 20–35 years were recruited. At the recruitment stage the participants performed ≥2 exercise sessions per week for the past 3 months and were not injured. None of them had any background in regular PJT or competitive sports with any kind of jumping exercises during the trial. During the study, five subjects withdrew due to personal reasons and consequently data from 47 subjects (mean ± standard deviation [SD]: age, 26.5 ± 3.9 years; body mass, 78 ± 9.2 kg; body height, 173.7 ± 7 cm; body mass index [BMI], 25.8 ± 2 kg/m^-2^; body fat, 18.8 ± 5.2%) were included in the final analysis (Fig 1). Using a random-numbers table and a simple type of randomization, subjects were randomly divided into four groups including TRS (no intra-set rest, n = 12), CLS10 (10 s intra-set rest, n = 11), CLS20 (20 s intra-set rest, n = 13), and CLS30 (30 s intra-set rest, n = 11) and their order to perform the measurements was random. After data collection, a random identification number was assigned to each subject by a person who was not involved in the study procedures, so that the authors had no access to information that could identify individual subjects.

The present research was conducted in accordance with the Declaration of Helsinki. All subjects signed written informed consent and were made aware of the risks associated. This study was approved by the Research Ethics committees of Baqiyatallah Hospital (IR.BMSU.BAQ.REC.1400.043) and was registered by the Thai Clinical Trials Registry (https://www.thaiclinicaltrials.org/show/TCTR20220210007).

### Procedures

This study used a between-subjects randomized design to examine the effects of PJT using the CLS structure with different rest intervals on physical fitness. The rest intervals including 0 (i.e., the TRS configuration), 10, 20, and 30 s were compared using four independent groups while other exercise prescription components were similar between them.

### Training program

Subjects were asked to achieve at least 7 h of sleep at night, and to avoid any other exercise training, except the PJT protocol during the study period. Before the beginning of training and testing, all subjects participated in a familiarization session covering all training and testing requirements. The training protocol comprised 2 sessions per week (Sundays and Wednesdays morning, at 9:00–11:00 am) of 4 exercises including countermovement jump, push-up jump, lateral skater jump, and incline push-up jump, across 6 weeks of training (Table 1). This training duration has been proposed to ensure neuromuscular adaptations without excessive fatigue or strain [15, 21]. All training sessions were completed in a gymnasium with rubber mat surface and subjects were required to jump for maximal effort. Given that subjects did not have any history of formal plyometrics, all training sessions were closely supervised, and particular attention was paid to demonstration and execution. Subjects performed a standardized warm-up at the beginning of each training session and were asked to refrain from any additional PJT or strength training throughout the study. All measurements were carried out 48 h before and after the training program at the same time of day. Of note, the four groups used the same total amount of rest during a given training session, with the only between-groups difference being the duration of the cluster rest.

**Table 1:** Training protocol.

Weeks	Sets	Groups: Cluster × Repetitions	Groups: Cluster rest (s)	Groups: Set rest (s)	Exercise rest (s)
1–2 3–4 5–6	3 4 5	TRS: 1 × 10–12*	TRS: 0	TRS: 90	90
		CLS10: 2 × 5–6	CLS10: 10	CLS10: 80	
		CLS20: 2 × 5–6	CLS20: 20	CLS20: 70	
		CLS30: 2 × 5–6	CLS30: 30	CLS30: 60	

TRS: traditional sets, CLS: cluster sets.

*Repetitions were 10 (2×5), and 12 (2×6) at weeks 1–2, and 3–6, respectively.

### Anthropometric measurements

Measurements were carried out in accordance with the guidelines described by the International Society for the Advancement of Kinanthropometry (ISAK) [24]. Standing height was measured to the nearest 0.5 cm. Body mass and the estimation of total body fat were determined by bioelectrical impedance analysis (BF511 Monitor, Omron Healthcare, Inc. Kyoto, Japan), based on the manufacturer’s guidelines, with a standard error of 3.5% for the estimation of body fat percentage. Reliability and validity of the instrument have been reported by previous studies [25, 26]. The BMI and fat-free mass (FFM) were also calculated from the standard equations (i.e., [body mass/height^-2^] and [body mass–body fat], respectively).

### Muscle strength test

Before the commencement of testing, a 10-min warm-up consisting of light-intensity movements and stretching, and two warm-up sets of exercises with no load was performed. Lower-body (i.e., back squat) and upper-body (i.e., chest press) dynamic strength was determined as the maximum weight lifted in a single and complete repetition (one repetition maximum [1RM]) in the same order as mentioned, according to a standardized 1RM testing protocol. The test has been shown to be valid and reliable in assessing muscle strength changes [27–29]. In brief, subjects performed a specific warm-up set with 5 repetitions carried out at ∼50% of subject’s perceived 1RM followed by 1 to 2 sets of 2–3 repetitions at a load corresponding to ∼60–80% 1RM. They then performed sets of 1 repetition of increasing load for 1RM determination [29]. This procedure was completed within 4 to 5 attempts and a rest period of at least 3 min was allotted between each attempt to avoid fatigue [30]. Proper lifting technique was demonstrated and practiced for each of the exercises. For evaluating grip strength on both hands, subjects were asked to maximally squeeze a hand dynamometer (78010, Lafayette, Inc. USA) without ancillary body movements, while the elbow was flexed to 90° at the side of the body. Two attempts were recorded (to the nearest 1 kg) with each hand, with at least 2 min of rest between them, and the highest records were used for the final analyses. In addition, at least 5 min of passive rest separated each strength test.

### Standing long jump (SLJ)

The SLJ is a practical, time-efficient, and low-cost test to assess lower-body muscular power, and its validity and reliability have been demonstrated [31]. Subjects performed the SLJ test two times, and the best record was used for data analysis. They were asked to execute a two-legged horizontal jump as far as possible out of a standing position with swinging arms and flexing knees. The distance from the beginning of the starting line to the most posterior aspect of the subject’s body was measured in cm by the same examiner.

### Change of direction speed (CODS) and linear sprinting speed tests

Validity and reliability of the tests has been previously reported [32, 33]. The CODS performance was determined by a 9-m shuttle run test on a hardcourt track marked with tapes and pylons. During the test, subjects sprint forward for 9 m and come back to the starting line. They passed the distance four times and had to touch the tapes at the end of the first three 9-m distances with either the left or the right hand. For assessing sprinting ability, a linear 20-m sprinting test was performed. In doing so, a standard hardcourt sprint track of 20 m length was prepared by setting up pylons and the starting and finishing lines were marked by white tapes on the floor. At the end of both tracks, there was enough space for subjects to continue moving until they stopped completely. Subjects started out of a standing position and had two attempts for each test with a 2–3 min rest period. A standard stopwatch was used to record time and the best time in seconds was used for data analysis. All measurements were conducted by the same examiner (blinded regarding subject’s groups allocation) who had practiced many times to become fully familiar with the device and to reduce the possible error of measurements.

### Local muscular endurance test

A validated and reliable back squat test for muscular endurance [34] was applied, where subjects performed a set at 60% 1RM for maximum repetitions. Once the back squat testing was finished, the chest press was conducted after a 4–5 min passive rest [30, 35]. Subjects were encouraged to perform as many repetitions as possible using the proper lifting techniques and the total number of proper repetitions performed was recorded.

### Perceived fatigue

This was determined using a new 11-point numerical scale ranging from zero to ten, called rating of fatigue (ROF), effective and valid in assessing changes in fatigue in a variety of contexts [36]. The ROF scale is a valid, simple, and sensitive instrument capable of tracking fatigue perception through exercise and recovery [36]. Twenty minutes after the first and last training session, each subject rated how fatigued they felt according to the numbers from 0 (referred to not fatigued at all) to 10 (referred to total fatigue and exhaustion). All subjects were thoroughly explained how to rate their perceived fatigue during the familiarization session.

### Diet control

**Fig 2:**
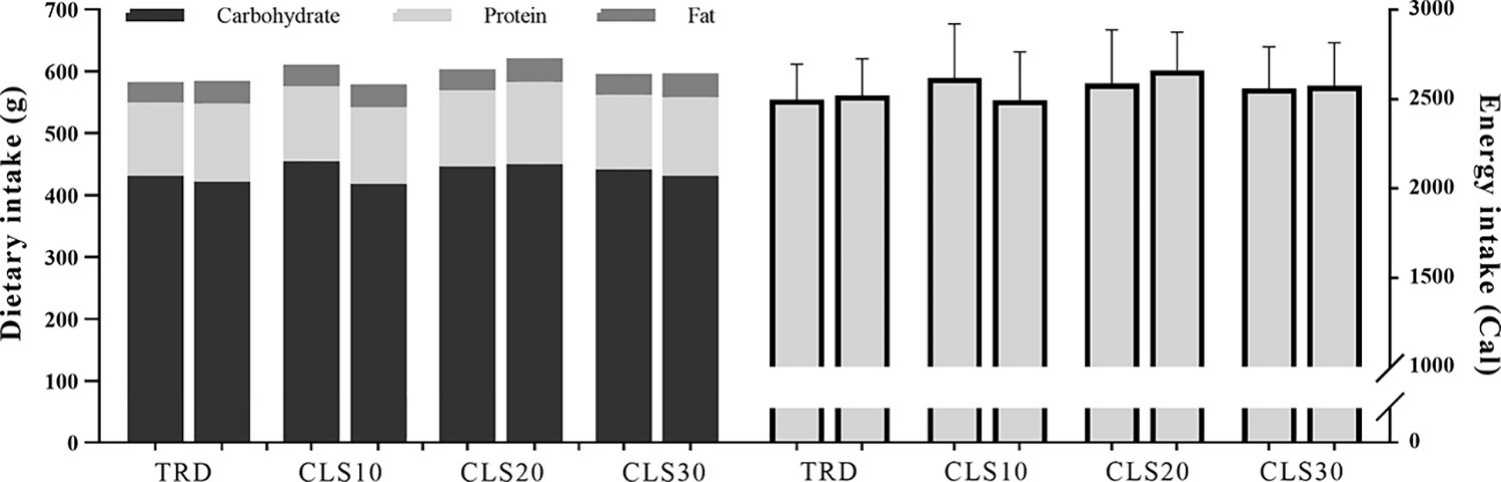
Mean of dietary and energy intake 48 h before pre-testing (left column for each group) and post-testing (right column for each group).

Diet was controlled as described by Arazi et al. (2022) [30]. Subjects were asked to maintain their habitual diets throughout the study. Written and verbal instructions were provided so that subjects could record the type and portion sizes of daily foods consumed 48 h before pre-test measurements. They also were instructed to mimic this diet 48 h before the post-test measurements. Dietary data analysis revealed no significant differences between groups before pre- and post-test sessions (Fig 2).

### Statistical analysis

The sample size was calculated using an a priori power analysis by G*Power software, version 3.1.9.4. Given a statistical power (1-ß error) of 0.9 (using the MANOVA statistical test: Repeated measures, within-between interaction) and a moderate effect size (ES) of 0.6, the total sample size resulted in 44 subjects. However, considering a 20% drop out rate, the minimal sample size was set at 52 subjects. The Statistical Package for Social Science (SPSS, v. 22^®^, Inc. Chicago, IL) was used to analyze data at an alpha level of *p* ≤ 0.05 for all tests. Pre- and post-intervention values for each dependent variable were analyzed to determine if the distributions were normal using the Shapiro-Wilk test. Data were analyzed using a 2 × 2 repeated measures ANOVA, and Bonferroni post hoc correction was applied to determine specific pairwise differences. Moreover, Cohen’s *d* was also calculated as ES statistic and the magnitudes were considered trivial (< 0.2), small (0.2 ≤ *d* < 0.5), medium (0.5 ≤ *d* < 0.8), and large (0.8 ≤) [30].

## Results

**Fig 3:**
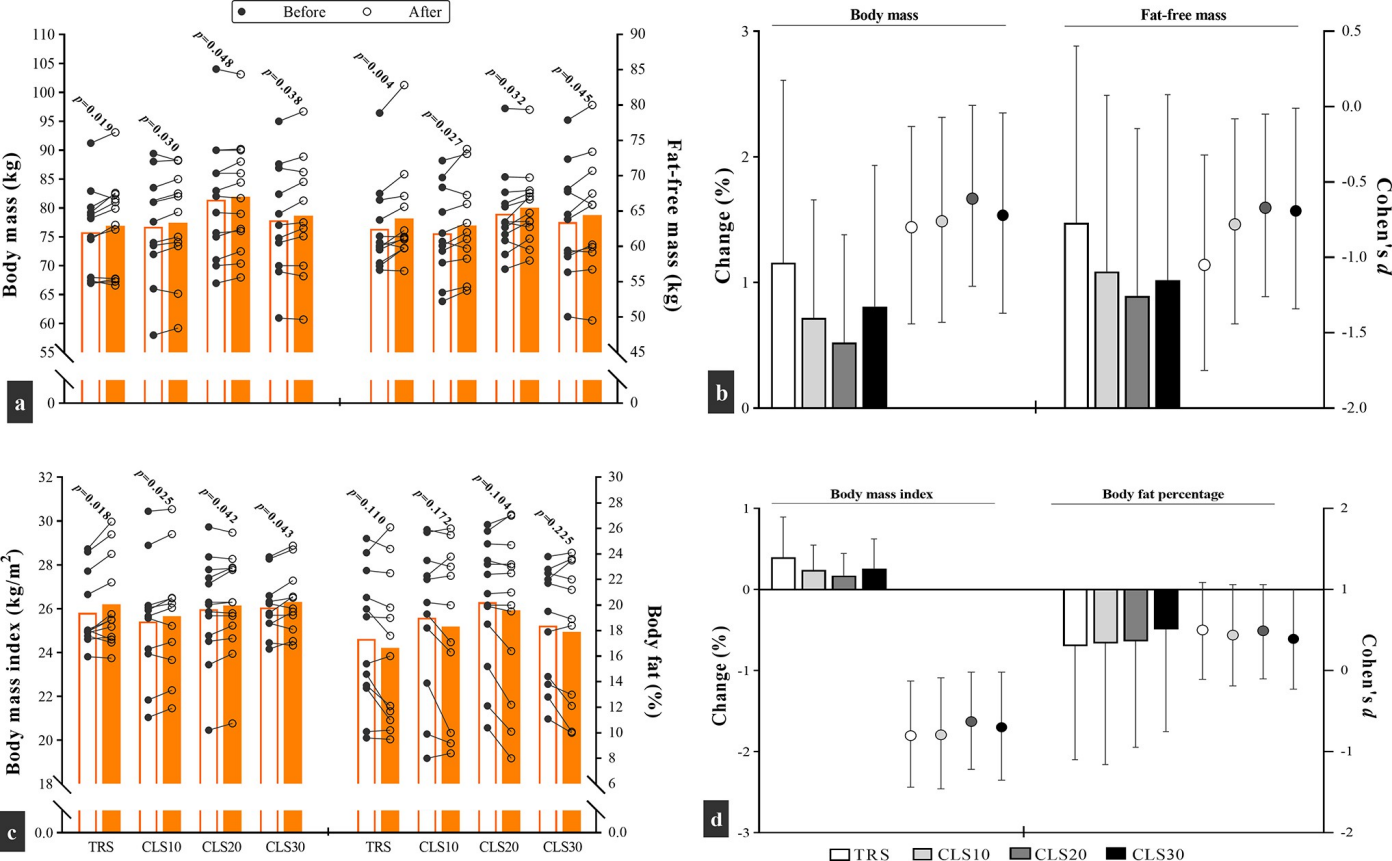
Changes in anthropometric outcomes after 6 weeks of plyometric jump training in traditional sets (TRS) group with no intra-set rest, and three cluster sets (CLS) groups with 10 (CLS10), 20 (CLS20), and 30 (CLS30) seconds intra-set rest. Fig a, c: white and orange columns denote outcomes results before and after the intervention. Moreover, each circle corresponds to a single subject. Fig b, d: the columns (percentage change) and circles (effect size) correspond to the left and right y axis, respectively.

As shown in Fig 3, the time × group interaction was not significant for body mass (F = 0.7; *p* = 0.558), FFM (F = 0.39; *p* = 0.759), BMI (F = 0.8; *p* = 0.498), and body fat percentage (F = 0.05; *p* = 0.985). However, the within-group comparisons indicated a significant reduction in body mass and BMI (ES = -0.8 to -0.6) and an increase in FFM (ES = -0.67 to -1.05) for all groups following the training program. In contrast, no significant change was found for body fat percentage (*p* > 0.05).

**Fig 4:**
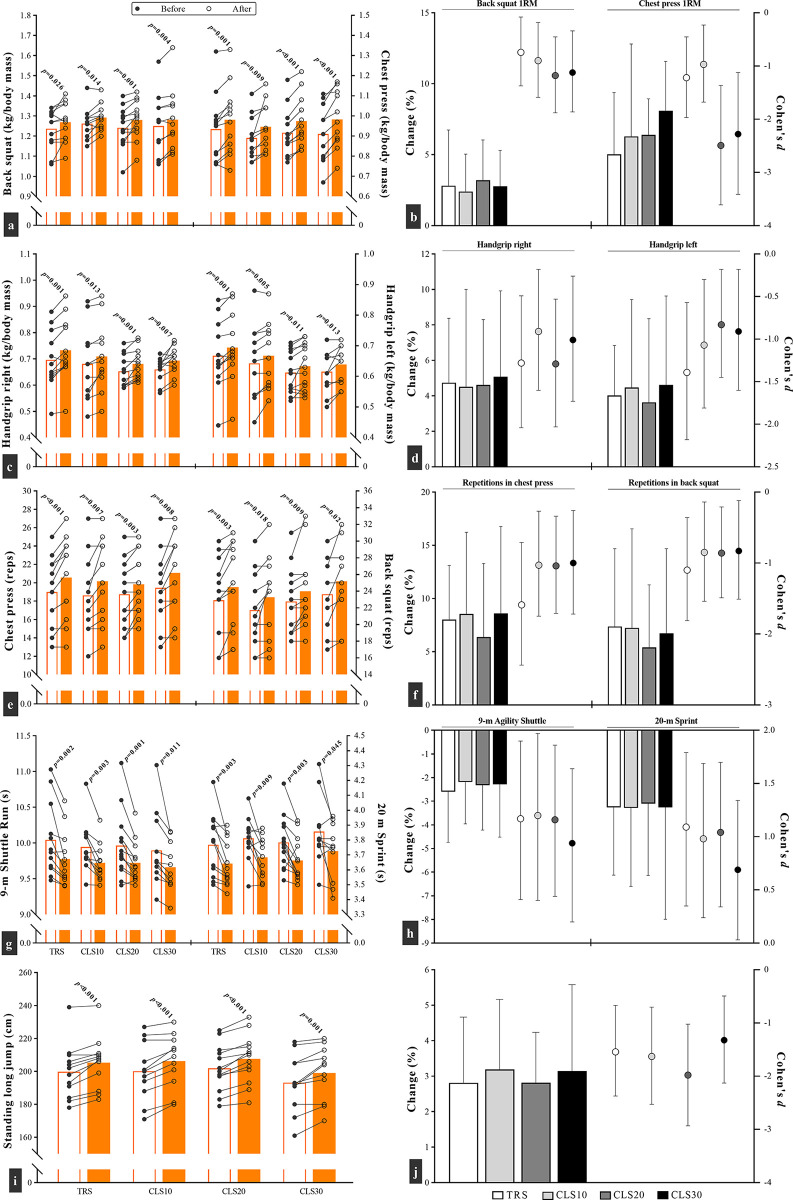
Changes in performance parameters after 6 weeks of plyometric jump training in traditional sets (TRS) group with no intra-set rest, and three cluster sets (CLS) groups with 10 (CLS10), 20 (CLS20), and 30 (CLS30) seconds intra-set rest. Fig a, c, e, g, i: white and orange columns denote outcomes results before and after the intervention. Moreover, each circle corresponds to a single subject. Fig b, d, f, h, j: the columns (percentage change) and circles (effect size) correspond to the left and right y axis, respectively.

Compared to their pre-training values, all four training groups showed significant improvements in the physical fitness components (Fig 4). Nonetheless, the time × group interaction was not significant for the squat (F = 0.14; *p* = 0.932) and chest press (F = 0.85; *p* = 0.475) 1RM, the right (F = 0.12; *p* = 0.946) and left (F = 0.9; *p* = 0.967) handgrip test, the squat (F = 0.13; *p* = 0.943) and chest press (F = 0.5; *p* = 0.69) repetitions to failure, the linear sprint (F = 0.01; *p* = 0.998) and CODS (F = 0.01; *p* = 0.961) records, and the SLJ (F = 0.07; *p* = 0.974).

**Fig 5:**
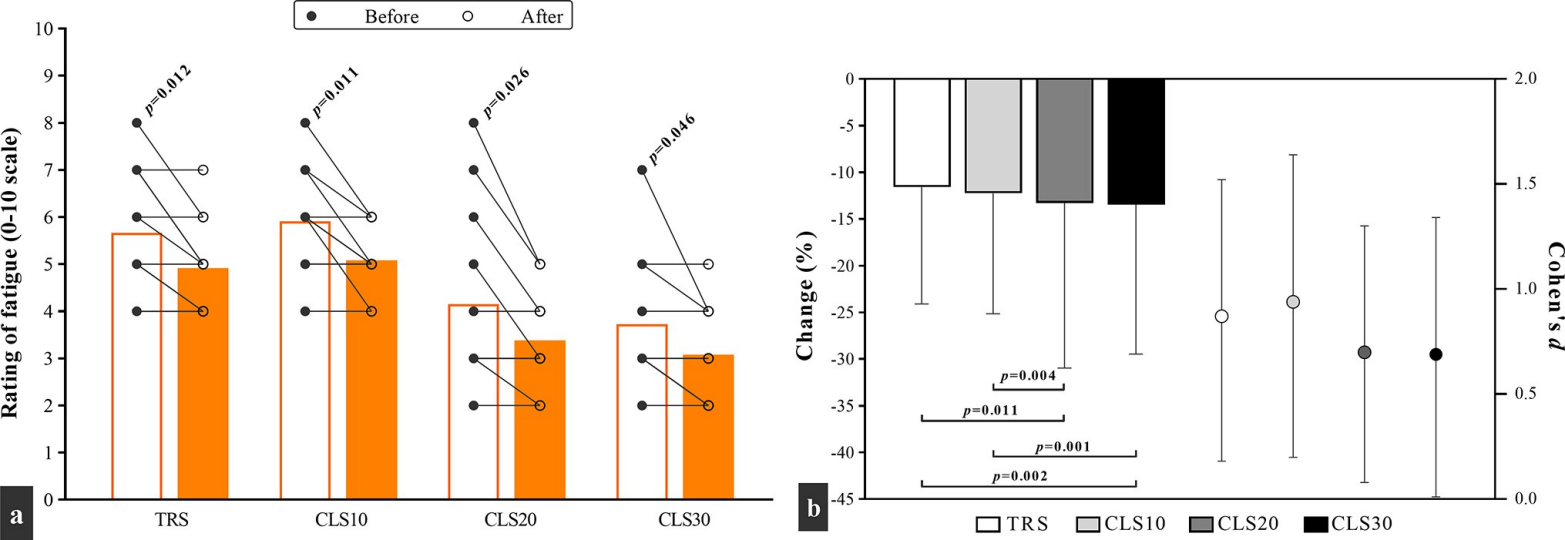
Changes in a) the rating of fatigue, and b) percentage change (the columns) and effect size (the circles), after 6 weeks of plyometric jump training in traditional sets (TRS) group with no intra-set rest, and three cluster sets (CLS) groups with 10 (CLS10), 20 (CLS20), and 30 (CLS30) seconds intra-set rest. Fig a: white and orange columns denote outcomes results before and after the intervention. Moreover, each circle corresponds to a single subject.

The ROF scores were significantly decreased (improved) from pre- to post-training in all four training groups (Fig 5). Although the time × group interaction was not significant (F = 0.07; *p* = 0.974), the CLS20 and CLS30 groups exhibited greater improvements compared to the TRS and CLS10 groups, based on the post-hoc Bonferroni test.

## Discussion

In recent years, PJT protocols have been modified by incorporating a brief intra-set rest period (i.e., 10 to 30 s) aimed toward lower exercise exertion. Such protocols, called CLS configuration, have been shown to be effective in enhancing sprint, jump and the CODS performance [21, 23]. In the present study, we compared the effects of different rest intervals during the CLS structure used in PJT on physical fitness factors in recreationally active men. In fact, to our knowledge, this is the first study that has investigated physical fitness adaptations to manipulating the intra-set rest period.

After 6 weeks, body composition variables changed in all groups, except body fat percentage. Although there was no statistically significant difference between groups, the TRS group showed large ESs for body mass and BMI reduction and FFM increase compared to medium ESs for the CLS groups. These observations agree with previous findings, indicating that PJT is associated with increasing FFM without changing fat mass [37]. Nonetheless, MacDonald et al. (2012) reported a significant gain in body fat percentage following 6 weeks of TRS plyometric training, changes probably attributed to modifications in subjects’ diet rather than the training protocol [17].

Fig 4A to 4D depicts the changes in relative strength measures after 6 weeks of training. The ES statistic obtained for the CLS20 (back squat, *d* = -1.2; chest press, *d* = -2.5) and CLS30 (back squat, *d* = -1.1; chest press, *d* = -2.3) groups was greater than the TRS (back squat, *d* = -0.7; chest press, *d* = -1.2) and CLS10 (back squat, *d* = -0.9; chest press, *d* = -1.0) groups. These results support previous studies indicating the effectiveness of plyometrics in enhancing maximal dynamic strength [17, 38, 39]. The increase in maximal dynamic strength may be associated to increased neuromuscular adaptations (e.g., increased recruitment of motor units) after PJT [6], and to the increase (~2%) in FFM observed in our study. However, the device used in the current study to measure body composition had a standard error of 3.5%. Therefore, findings should be interpreted with a degree of caution.

A novel finding in the present study was that all groups significantly improved local muscular endurance (maximum number of repetitions at 60% of 1RM) in chest press (6.4–8.6%) and back squat (5.4–7.4%), whereas the respective ES values were high, with a greater magnitude for the TRS group when compared to the other three groups (Fig 4E and 4F). Of note, we re-adjusted the load in the post-test assessment according to the new (increased) 1RM of the participants, thus reducing the contribution of increased maximal strength to the muscle endurance test results. Improved local muscular endurance may be related to an enhanced oxidative and buffering capacity, increase in capillarization and mitochondrial volume, and improved metabolic enzyme activity [40]. However, the scarce number of studies related to PJT and muscle endurance make difficult the discussion of current findings with comparative data. Nonetheless, a systematic review indicated that PJT may increase repeated sprint ability [41], a physical fitness trait related to muscle energy supply mechanisms [42], including phosphocreatine hydrolysis, anaerobic glycolysis and oxidative metabolism, intramuscular disposal of metabolic by-products (e.g., hydrogen ions), activation of muscle and inter-muscle recruitment strategies, stiffness regulation, and muscle damage. PJT can improve some of these factors, including stiffness [43], muscle activation [44], and muscle damage [45]. However, it remains to be elucidated the mechanisms through which some forms of PJT, particularly those requiring reduced inter-repetition rest [46] (e.g., as the TRS group in our study), may improve endurance.

All groups significantly enhanced the CODS and linear sprint performance, and the respective ESs were moderate to high (Fig 4G and 4H). Although some authors have reported no effect of PJT on 20 m sprint [47] and the CODS [48, 49] records, our findings add consistently to previous works showing that sprint ability and the CODS performance improved [21, 23] following 6 weeks of PJT using a CLS approach. These improvements might be attributed to increased muscle strength, particularly back squat strength, as it is highly correlated to sprint [50] and CODS [51]. Additional factors related to sprint ability and CODS performance improvement may include increased motor unit recruitment, in line with greater muscle power output and force development ability [15, 21], lower ground contact times as a result of high muscular force output [21, 52], increased rate of force development, and the increased efficiency in using the stretch-shortening cycle (SSC) during ballistic tasks [11]. Nonetheless it remains to be elucidated if such speculated physiological mechanism occurs (and its magnitude) after different cluster set configurations.

Another important change observed in this study was the increase in the SLJ distance (2.8–3.2%) in all four groups after the training period (Fig 4I). The calculated ES statistics ranged between 1.3 and 2, while the greater magnitude was related to the CLS20 group (Fig 4J). Numerous studies have found significant improvements in jumping performance as a result of short-term PJT [8, 21, 23, 52–54], supporting our results and the principle of training specificity. There are several explanations for the positive effects of plyometrics, mainly related to neural adaptations, including changes in muscle structure and individual fiber mechanics, enhanced neuro-muscular coordination, changes in mechanical stiffness properties of tendons and the greater efficiency to utilize the muscles SSC [21, 52]. Regardless of these reasons, part of the improvements may be due to the increase in FFM of subjects in all groups and subsequent increase in muscular power output and the rate of force development.

Regarding ROF differences between groups (Fig 5), our results revealed a significant decrease in ROF scores in all groups. In fact, the ROF score in all groups was lower after training, even considering that the number of sets and repetitions were greater in the last training session compared to the first week. Of note, subjects who performed PJT with a 20 or 30 s intra-set rest interval reported the lower ROF values, and this result seems independent from the effect induced by PJT. Depletion of muscle creatine content [55], pH drop due to lactate accumulation, and muscle glycogen depletion [56] can be among the possible metabolic causes of exercise-induced fatigue. By continuing to jump during plyometric exercise, tension develops in muscles and they provide the necessary force to continue exercise through increasing motor unit recruitment and firing frequency [56]. Greater motor unit recruitment, in turn, can increase more signals to the sensory cortex [56, 57] and subsequently increase the perceptions of exertion and fatigue. However, it is speculative to discuss more about these metabolic and neuromotor factors because we did not assess them, thus future studies should solve this.

Strengths of this study include its randomized design, inclusion of non-athlete subjects to overcome the possible effects of previous exercise experience, and control of dietary intake during the days before pre- and post-test measurements. Despite these strengths, the current study has some potential limitations that should be taken into account when interpreting the results. Although all subjects were instructed to maintain their regular daily diet and physical activity throughout the study, the exact amount of these factors was not controlled separately. Furthermore, the use of hand-held chronometers to measure linear sprint and CODS time might have reduced measurement accuracy compared to more advanced equipment (e.g., electronic timing systems).

## Conclusions

A six-week upper- and lower-body PJT program improved physical fitness and anthropometric measures in recreationally active young men, regardless of whether an intra-set rest of 0, 10, 20, or 30 s is used. However, the inclusion of 20 or 30 s rest into the sets (i.e., the CLS20 and CLS30 configurations, respectively) reduced exercise-induced fatigue perception. Current findings provide novel and practical relevance to design optimal plyometric training protocols using the CLS or TRS configuration for the improvement of physical fitness and body composition, in line with reduced perceived fatigue, a key element for long-term physical activity habits.

## Supporting information

S1 File PLOS’ questionnaire on inclusivity in global research. (DOCX)

S2 File CONSORT checklist. (PDF)

S3 File The study protocol. (PDF)

S4 File Excel datasets for dietary data analysis. Fig 2 is released from these datasets.
(XLSX)

S5 File Excel datasets for anthropometric and physical fitness factors analysis. Figs 3–5 are released from these datasets.
(XLSX)
