# Supplementary material for: Effects of different cluster-set rest intervals during plyometric-jump training on measures of physical fitness: A randomized trial
Source: PLoS One. 2023 Oct 4;18(10):e0285062. doi: 10.1371/journal.pone.0285062 (PMC10550132; doi:10.1371/journal.pone.0285062)
Supplement: S3 File — (PDF) [file pone.0285062.s003.pdf]

« بسمه تعالی »

دانشگاه علوم پزشکی بقیه الله (عج)

شناسنامه طرح تحقیقاتی

عنوان:

مقایسه اثر تمرینات پلایومتریک خوشه‌ای با فواصل استراحتی مختلف بر ترکیب بدن، قدرت و توان عضلانی، سرعت حرکت و چابکی پرسنل نظامی

Title:

Comparison of cluster sets plyometric training with different rest intervals on body composition, muscular strength and power, speed of movement, and agility in military forces

سرپرست اول: دکتر بهزاد بازگیر

سرپرست دوم: دکتر حسین شیروانی

مجری: بهزاد طاعتی مقدم

The first supervisor: Behzad Bazgir, Ph.D
The second supervisor: Hossein Shirvani, Ph.D
Presented by: Behzad Taaty Moghadam

مرکز ارائه‌دهنده: مرکز تحقیقات فیزیولوژی ورزش

Supported by: Sports Physiology Research Center

۱ – تعریف واژه‌ها:

تمرینات پلایومتریک: نوعی از تمرینات با شدت بالا هستند که توانایی عضلات برای تولید نیرو در واحد زمان را از طریق بهبود چرخه کشش-کوتاه شدن ارتقا می‌دهند و در نتیجه، توانایی فرد برای انجام کارهای با توان بالا افزایش پیدا می‌کند [۲]. در پژوهش حاضر، پروتکل تمرینات پلایومتریک به دو روش سنتی و خوشه‌ای انجام خواهد شد.

چرخه کشش-کوتاه شدن: این چرخه زمانی روی می‌دهد که بلافاصله پس از طویل شدن عضله فعال در مرحله برون‌گرای حرکت، یک انقباض عضلانی درون‌گرا (کوتاه شدن) انجام شود که به‌طور معمول در تمرینات پلایومتریک مشاهده می‌شود [۷].

تمرینات خوشه‌ای: ساختاری از ست‌های تمرینی است که در آن یک فاصله استراحتی مشخص ۱۰ تا ۳۰ ثانیه‌ای بین برخی از تکرارهای یک ست تمرینی گنجانده می‌شود [۵]. در پژوهش حاضر، فواصل استراحتی مختلف بین خوشه‌های تمرینی با هم مقایسه می‌شوند.

ترکیب بدن: ترکیب بدن به‌منظور توضیح درصد چربی، عضله، استخوان و آب در بدن مورد استفاده قرار می‌گیرد [۶]. در پژوهش حاضر، درصد و توده چربی بدن با استفاده از کالیپر استاندارد اندازه‌گیری شده و مقادیر توده بدون چربی و شاخص توده بدن (BMI) آزمودنی‌ها نیز محاسبه می‌شود.

قدرت بیشینه: حداکثر وزنه‌ای را که یک عضله یا گروه عضلانی، فقط برای یک بار می‌تواند جابجا کند، قدرت بیشینه آن عضله یا گروه عضلانی در نظر گرفته می‌شود [۳]. در پژوهش حاضر، قدرت بیشینه عضلات بالا و پایین‌تنه مورد اندازه‌گیری قرار می‌گیرد.

توان عضلانی: بیش‌ترین نیروی تولیدی در واحد زمان به‌عنوان توان در نظر گرفته می‌شود. در پژوهش حاضر، از آزمون پرش طول درجا به منظور ارزیابی توان عضلات پایین‌تنه آزمودنی‌ها استفاده می‌شود.

سرعت حرکت: طی یک مسافت مشخص در کم‌ترین زمان ممکن به‌عنوان سرعت حرکت تعریف می‌شود. در این پژوهش، آزمون دوی ۲۰ متر برای سنجش این متغیر استفاده خواهد شد.

چابکی: توانایی تغییر مسیر ناگهانی با کم‌ترین افت سرعت و با حفظ تعادل به‌عنوان چابکی تعریف می‌شود. در پژوهش حاضر، از آزمون رفت و برگشت ۹×۴ متر به‌منظور اندازه‌گیری این متغیر استفاده خواهد شد.

1- Definition of words:

Plyometric training: A type of power exercises that improves the ability of muscles to produce force per unit of time by improving the stretch-shortening cycle, and as a result, increases the ability of a person to perform high-powered tasks [2]. In the present study, the protocol of plyometric training will be performed in two methods including traditional and cluster.

Stretch-shortening cycle: This cycle that is typically observed in plyometric exercises, occurs when a concentric muscle contraction (shortening) occurs immediately after the lengthening of the active muscle in the eccentric phase of the action [7].

Cluster training: A structure of training sets in which a specified rest interval of 10 to 30 s is included between some repetitions of a training set [5]. In the present study, different rest intervals will be compared between training clusters.

Body composition: Body composition is used to determine the percentage of fat, muscle, bone and water in the body [6]. In the present study, body fat percentage will be measured using a standard caliper, and the subjects' fat-free mass and body mass index (BMI) will also be calculated.

Maximum strength: The maximum force that a muscle or muscle group can generate, just for one time [3]. In the present study, the maximum strength of upper- and lower-body muscles will be measured.

Muscular power: The maximum force per unit of time is considered as power. In the present study, the standing long jump test will be used to measure the subjects' lower-body muscular power.

Speed of movement: It is defined as the speed of movement over a certain distance in the shortest possible time. In this research, 20 m sprint test will be used to measure this variable.

Agility: The ability to change direction quickly with the least loss of speed and with maintaining balance. In this research, a 9-m shuttle run test will be used to measure this variable.

۲ - هدف کلی:

مقایسه اثر تمرینات پلایومتریک خوشه‌ای با فواصل استراحتی مختلف بر ترکیب بدن و برخی عوامل آمادگی جسمانی پرسنل نظامی

2- General objective

Comparison of cluster sets plyometric training with different rest intervals on body composition and some physical fitness factors in military forces

۳ – اهداف جزیی:

۱. بررسی و مقایسه اثر یک دوره تمرینات پلایومتریک خوشه‌ای با فواصل استراحتی مختلف بر وزن بدن پرسنل نظامی
۲. بررسی و مقایسه اثر یک دوره تمرینات پلایومتریک خوشه‌ای با فواصل استراحتی مختلف بر BMI پرسنل نظامی
۳. بررسی و مقایسه اثر یک دوره تمرینات پلایومتریک خوشه‌ای با فواصل استراحتی مختلف بر درصد چربی بدن پرسنل نظامی
۴. بررسی و مقایسه اثر یک دوره تمرینات پلایومتریک خوشه‌ای با فواصل استراحتی مختلف بر توده بدون چربی بدن پرسنل نظامی
۵. بررسی و مقایسه اثر یک دوره تمرینات پلایومتریک خوشه‌ای با فواصل استراحتی مختلف بر قدرت عضلانی پرسنل نظامی
۶. بررسی و مقایسه اثر یک دوره تمرینات پلایومتریک خوشه‌ای با فواصل استراحتی مختلف بر توان عضلانی پرسنل نظامی
۷. بررسی و مقایسه اثر یک دوره تمرینات پلایومتریک خوشه‌ای با فواصل استراحتی مختلف بر سرعت حرکت پرسنل نظامی
۸. بررسی و مقایسه اثر یک دوره تمرینات پلایومتریک خوشه‌ای با فواصل استراحتی مختلف بر چابکی پرسنل نظامی

3- Specific objectives

1. Investigation and comparison of a period of cluster sets plyometric training with different rest intervals on body mass of military forces
2. Investigation and comparison of a period of cluster sets plyometric training with different rest intervals on BMI of military forces
3. Investigation and comparison of a period of cluster sets plyometric training with different rest intervals on body fat percentage of military forces
4. Investigation and comparison of a period of cluster sets plyometric training with different rest intervals on fat-free mass of military forces
5. Investigation and comparison of a period of cluster sets plyometric training with different rest intervals on muscle strength of military forces

6. Investigation and comparison of a period of cluster sets plyometric training with different rest intervals on muscular power of military forces
7. Investigation and comparison of a period of cluster sets plyometric training with different rest intervals on speed of movement of military forces
8. Investigation and comparison of a period of cluster sets plyometric training with different rest intervals on agility of military forces

۴ – اهداف کاربردی:

طراحی و اجرای یک پروتکل بهینه تمرینات پلایومتریک با کارایی بالا برای بهبود عوامل ترکیب بدن و افزایش سرعت حرکت، قدرت عضلانی، توان عضلانی و چابکی نیروهای مسلح

4- Applied objectives

Designing and performing an optimal plyometric training protocol with high efficiency to improve body composition factors and increase sprint, muscle strength, muscular power and agility in military forces

۵- فرضیات:

۱. فواصل استراحتی مختلف به‌کار گرفته شده در تمرینات پلایومتریک خوشه‌ای تاثیر متفاوتی بر وزن بدن پرسنل نظامی دارد.

۲. فواصل استراحتی مختلف به‌کار گرفته شده در تمرینات پلایومتریک خوشه‌ای تاثیر متفاوتی بر BMI پرسنل نظامی دارد.

۳. فواصل استراحتی مختلف به‌کار گرفته شده در تمرینات پلایومتریک خوشه‌ای تاثیر متفاوتی بر درصد چربی بدن پرسنل نظامی دارد.

۴. فواصل استراحتی مختلف به‌کار گرفته شده در تمرینات پلایومتریک خوشه‌ای تاثیر متفاوتی بر توده بدون چربی بدن پرسنل نظامی دارد.

۵. فواصل استراحتی مختلف به‌کار گرفته شده در تمرینات پلایومتریک خوشه‌ای تاثیر متفاوتی بر قدرت عضلانی پرسنل نظامی دارد.

۶. فواصل استراحتی مختلف به‌کار گرفته شده در تمرینات پلایومتریک خوشه‌ای تاثیر متفاوتی بر توان عضلانی پرسنل نظامی دارد.

۷. فواصل استراحتی مختلف به‌کار گرفته شده در تمرینات پلایومتریک خوشه‌ای تاثیر متفاوتی بر سرعت حرکت پرسنل نظامی دارد.

۸. فواصل استراحتی مختلف به‌کار گرفته شده در تمرینات پلایومتریک خوشه‌ای تاثیر متفاوتی بر چابکی پرسنل نظامی دارد.

5- Hypotheses

1. Different rest intervals used in cluster sets plyometric training have a different effect on body mass of military forces
2. Different rest intervals used in cluster sets plyometric training have a different effect on BMI of military forces

3. Different rest intervals used in cluster sets plyometric training have a different effect on body fat percentage of military forces<br>4. Different rest intervals used in cluster sets plyometric training have a different effect on fat-free mass of military forces<br>5. Different rest intervals used in cluster sets plyometric training have a different effect on muscle strength of military forces<br>6. Different rest intervals used in cluster sets plyometric training have a different effect on muscular power of military forces<br>7. Different rest intervals used in cluster sets plyometric training have a different effect on sprint of military forces<br>8. Different rest intervals used in cluster sets plyometric training have a different effect on agility of military forces
**۶- نوع مطالعه :**<br>مطالعه نیمه‌تجربی حاضر یک کارآزمایی تصادفی شده مقطعی است.<br>**6- The type of study**<br>The present study is a quasi-experimental, randomized cross-sectional trial.
**۷- جامعه مورد مطالعه :**<br>جامعه مورد مطالعه پژوهش حاضر عبارت از کلیه نیروهای نظامی سالم استان گیلان می‌باشند.<br>**7- Study population**<br>The population of the present study consists of all healthy military forces of the Guilan state.
**۸- حجم نمونه و روش محاسبه آن :**<br>حجم نمونه با استفاده از نرم‌افزار آماری G*Power 3.1 محاسبه شد [۴]. با استفاده از این نرم‌افزار برای آزمون آماری F با چهار گروه مستقل، در سطح آماری ۰/۰۵، توان آماری بیش‌تر از ۸۵ درصد و اندازه اثر بالا، حجم کل نمونه برابر با ۴۴ نفر به‌دست آمد (شکل ۱). با وجود این، با در نظر گرفتن نرخ ۲۰ درصدی خروج احتمالی آزمودنی‌ها از پژوهش، حداقل حجم نمونه ۵۲ نفر تعیین شد.<br>**8- Sample size and its calculation method**<br>The sample size was calculated using G*Power 3.1 statistical software [4]. By using this software for the F statistic test with four independent groups, at the statistical level of 0.05, the statistical power of 90% and the effect size of 0.6, the total sample size is equal to 44 subjects (Figure 1). However, considering a 20% drop out rate, the minimal sample size was set at 52 subjects.

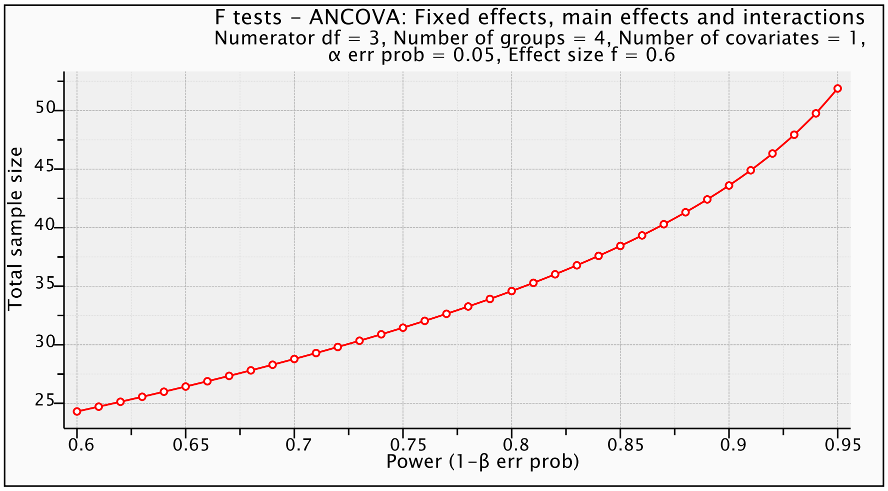

شکل ۱- محاسبه حجم کل نمونه برای آزمون F

Figure 1- The calculation of the total sample size for the F test

۹- روش تجزیه و تحلیل داده ها:

پس از بررسی طبیعی بودن توزیع داده‌ها با استفاده از آزمون شاپیرو ویلک (Shapiro–Wilk test)، از آزمون آماری تحلیل واریانس یک‌راهه (ANOVA) برای بررسی تفاوت‌های مشاهده شده بین گروه‌های پژوهش استفاده می‌شود. همچنین، برای بررسی تغییرات درون‌گروهی در هر یک از گروه‌های پژوهش (تفاوت‌های پس‌آزمون نسبت به پیش‌آزمون) از آزمون t وابسته استفاده خواهد شد. داده‌ها به‌صورت میانگین ± انحراف معیار ارائه شده و از نرم‌افزار آماری SPSS نسخه ۲۲ استفاده خواهد شد. همچنین، سطح معنی‌داری آزمون‌ها کم‌تر از ۰/۰۵ در نظر گرفته می‌شود.

9- Statistical analysis

After determining the normality of the data distribution using the Shapiro-Wilk test, an analysis of covariance (ANCOVA) test will be used to compare the observed differences between the groups. Additionally, to examine intra-group changes in each group (post-test differences compared to pre-test), dependent t-tests will be used. The data will be presented as mean ± standard deviation, and the statistical software SPSS version 22 will be used. Furthermore, the significance level of the statistical tests will be considered less than 0.05.

۱۰- ملاحظات اخلاقی:

۱. شرکت در این پژوهش کاملا داوطلبانه بوده و آزمودنی‌ها در هر مرحله از روند اجرای پژوهش می‌توانند از ادامه همکاری انصراف دهند.

۲. اهداف، روند اجرای پژوهش و فواید و خطرات احتمالی شرکت در پژوهش به‌طور کامل برای آزمودنی‌ها شرح داده شده و فرم رضایت شرکت در پژوهش از تمامی آنان اخذ خواهد شد.

۳. فواید شرکت در این پژوهش برای آزمودنی‌ها عبارت از اندازه‌گیری وزن بدن، مقدار چربی و عضله بدن، سرعت حرکت، چابکی، قدرت و توان عضلانی و محاسبه میزان انرژی دریافتی روزانه خواهد بود.
٤. این پژوهش زیر نظر مرکز تحقیقات فیزیولوژی ورزش دانشگاه علوم پزشکی بقیه الله (عج) انجام شده و کد اخلاق قابل پیگیری اخذ خواهد شد.
۵. کلیه اطلاعات به‌دست آمده از آزمودنی‌ها نزد محقق محفوظ بوده و اصل امانت‌داری به‌طور کامل اجرا خواهد شد.

10- Ethical considerations

1. Participation in this study is entirely voluntary, and the participants can withdraw from the collaboration at any phase of the research process.
2. The objectives, implementation process, and potential benefits and risks of participating in the research will be fully described to the participants, and a consent form will be obtained from all of them.
3. The benefits of participating in this study for the participants include measuring body mass, body fat percentage and muscle mass, speed of movement, agility, muscle strength, and calculating daily energy intake.
4. This study is being conducted under the supervision of the Exercise Physiology Research Center at Baqiyatallah University of Medical Sciences, and a traceable ethics code will be obtained.
5. All information obtained from the participants will be kept confidential by the researcher, and the principle of confidentiality will be fully implemented.

۱۱- مشکلات و محدویت‌ها:

محدودیت‌های پژوهش حاضر عبارت است از:

۱. شرایط روانی و انگیزشی آزمودنی‌ها
۲. تفاوت‌های فردی موجود بین آزمودنی‌ها
۳. کنترل دقیق و مستقیم تغذیه آزمودنی‌ها
٤. خواب شبانه و استراحت آزمودنی‌ها

11- Challenges and limitations

The limitations of this research include:

1. Psychological and motivational conditions of the subjects.
2. Individual differences among the subjects.
3. Accurate and direct control of the subjects' nutrition.
4. Subjects' nocturnal sleep and rest.

۱۲- روش انجام طرح (شیوه اجرایی مراحل طرح و چگونگی جمع آوری اطلاعات):

بر اساس محاسبات انجام شده برای تعیین حجم نمونه پژوهش حاضر، تعداد ۵۲ نفر از نیروهای نظامی شهر رشت به‌صورت نمونه در دسترس بر اساس معیارهای ورود به پژوهش از قبیل محدوده سنی ۲۰ تا ۳۵ سال، داشتن تمرینات منظم ورزشی به‌منظور برخورداری از سطح آمادگی اولیه جهت شرکت در تمرینات پلایومتریک، عدم ابتلا به بیماری یا آسیب، عدم مصرف دخانیات برای شرکت در پژوهش انتخاب می‌شوند. همچنین، معیارهای خروج از پژوهش عبارت از بروز آسیب یا بیماری در دوره اجرای تمرینات،

عدم همکاری آزمودنی در اجرای درست تمرینات، غیبت بیش از یک جلسه در دوره اجرای پروتکل تمرینات پلایومتریک می‌باشد. توضیحات کامل در خصوص هدف پژوهش، روند اجرا و فواید و خطرات احتمالی در اختیار آزمودنی‌ها قرار گرفته و فرم رضایت شرکت داوطلبانه در پژوهش حاضر از آنان اخذ خواهد شد. سپس، داوطلبان به‌طور تصادفی به چهار گروه ۱۳ نفری شامل پلایومتریک سنتی، پلایومتریک ۱۰ (استراحت ۱۰ ثانیه‌ای)، پلایومتریک ۲۰ (استراحت ۲۰ ثانیه‌ای) و پلایومتریک ۳۰ (استراحت ۳۰ ثانیه‌ای) تقسیم می‌شوند.

12- Challenges and limitations

Based on the calculations performed to determine the sample size for the present study, a total of 52 military forces from the city of Rasht will be selected based on inclusion criteria such as age range of 20 to 35 years, having regular exercise training in order to have a basic level of physical fitness to participate in the plyometric training protocol, no illness or injury, and no use of tobacco. In addition, the exclusion criteria include injury or illness during the training period, improper execution of exercises, and absence from more than one session during the training protocol. A complete explanation of the research objectives, implementation process, and potential benefits and risks will be provided to the participants, and a voluntary consent form will be obtained from them. Then, volunteers will be randomly divided into four groups of 13 subjects, including traditional plyometric training, plyometric 10 (with 10 s of intra-set rest), plyometric 20 (with 20 s of intra-set rest), and plyometric 30 (with 30 s of intra-set rest).

۱۲-۱- پروتکل تمرینات پلایومتریک

پیش از آغاز دوره تمرینات، آزمودنی‌ها طی یک جلسه با نحوه انجام حرکات و سایر نکات لازم برای اجرای صحیح پروتکل تمرینات پلایومتریک آشنا خواهند شد. از نقطه نظر فیزیولوژیکی و روانشناسی، شش هفته تمرینات پرش عمقی با شدت بالا مدت مطلوبی است تا سیستم عصبی مرکزی بدون بروز خستگی بیش از حد یا آسیب، تحت استرس قرار بگیرد [۱]. بنابراین، در پژوهش حاضر، تمرینات دو جلسه در هفته به‌مدت شش هفته انجام خواهد شد. تمرینات پایین‌تنه شامل دو حرکت پرش متقاطع و پرش جانبی تک‌پا و تمرینات بالاتنه شامل دو حرکت شنا سوئدی پرشی و شنا سوئدی پرشی شیب مثبت خواهد بود.

12-1- Plyometric training protocol

Prior to the start of the training period, participants will become familiar with the proper execution of plyometric exercises and other necessary points. From a physiological and psychological point of view, six weeks of stretch-shortening cycle training is desirable for the central nervous system to undergo stress without excessive fatigue or strain [1]. Therefore, in the present study, two sessions of plyometric training will be performed per week for six weeks. Lower-body exercises include countermovement jump and lateral skater jump, and upper-body exercises include push-up jump and incline push-up jump.

پروتکل تمرینات پلایومتریک برای گروه‌های مختلف در پژوهش حاضر در جدول ۱ ارائه شده است.

جدول ۱- پروتکل تمرینات پلایومتریک شش هفته‌ای برای گروه‌های پژوهش

گروه‌های پژوهش	تعداد ست‌ها و تکرارها	استراحت بین خوشه‌ها (ثانیه)	استراحت بین ست‌ها (ثانیه)	استراحت بین حرکات (ثانیه)	حجم کل تکرارها و استراحت در هر جلسه
پلایومتریک سنتی	هفته ۲-۱: ۳ ست ۱۰ تکراری<br>هفته ۴-۳: ۴ ست ۱۲ تکراری<br>هفته ۶-۵: ۵ ست ۱۲ تکراری	صفر	۹۰	۹۰	هفته ۲-۱: ۳۰ تکرار برای هر حرکت، استراحت کل برابر ۱۸۰ ثانیه

پلایومتریک ۱۰	هفته ۲-۱: ۳ ست ۱۰ تکراری به‌صورت ۲ خوشه ۵ تکراری در هر ست	۱۰	۸۰		هفته ۴-۳: ۴۸ تکرار برای هر حرکت، استراحت کل برابر ۱۸۰ ثانیه<br>هفته ۶-۵: ۶۰ تکرار برای هر حرکت، استراحت کل برابر ۱۸۰ ثانیه
پلایومتریک ۲۰	هفته ۴-۳: ۴ ست ۱۲ تکراری به‌صورت ۲ خوشه ۶ تکراری در هر ست	۲۰	۷۰		
پلایومتریک ۳۰	هفته ۶-۵: ۵ ست ۱۲ تکراری به‌صورت ۲ خوشه ۶ تکراری در هر ست	۳۰	۶۰		

The plyometric training protocol for different groups is presented in Table 1.

Table 1- The plyometric training protocol.

Groups	Sets and repetitions	Cluster rest (s)	Set rest (s)	Exercise rest (s)	Total repetitions and rest per session
Traditional	**Week 1-2:** 3 sets × 10 reps<br>**Week 3-4:** 4 sets × 12 reps<br>**Week 5-6:** 5 sets × 12 reps	0	90	90	**Week 1-2:** 30 reps for each exercise, total rest of 180 s<br>**Week 3-4:** 48 reps for each exercise, total rest of 180 s<br>**Week 5-6:** 60 reps for each exercise, total rest of 180 s
Cluster-set 10	**Week 1-2:** 3 sets × 10 reps, as 2 clusters of 5 reps per set<br>**Week 3-4:** 4 sets × 12 reps, as 2 clusters of 6 reps per set<br>**Week 5-6:** 5 sets × 12 reps, as 2 clusters of 6 reps per set	10	80		
Cluster-set 20		20	70		
Cluster-set 30		30	60		

قبل و بعد از دوره تمرینات، متغیرهای ترکیب بدن و عوامل آمادگی جسمانی آزمودنی‌ها که به‌شرح زیر می‌باشد اندازه‌گیری خواهد شد تا تاثیر دوره تمرینات پلایومتریک و فواصل استراحتی مختلف مشخص گردد.

Before and after the training period, body composition and physical fitness factors will be measured as follows to determine the effect of plyometric training and different rest intervals.

الف) متغیرهای ترکیب بدن

وزن بدن: آزمودنی‌ها با بر تن داشتن حداقل لباس برای چند ثانیه و بدون حرکت روی ترازوی استاندارد (Seca، مدل ۸۱۳ ساخت کشور آلمان) ایستاده و وزن بدن آن‌ها با دقت ۰/۱ کیلوگرم اندازه‌گیری و ثبت خواهد شد.

درصد و توده چربی بدن: ارزیابی درصد چربی بدن آزمودنی‌ها با اندازه‌گیری چربی زیرپوستی آنان در سه نقطه از سمت راست بدن (سینه، شکم و ران) و با استفاده از دستگاه کالیپر استاندارد (Lafayette، مدل 01127A، ساخت کشور آمریکا) انجام می‌شود. همچنین، با ضرب کردن درصد چربی آزمودنی‌ها در وزن بدن آن‌ها، مقدار چربی بدن بر حسب کیلوگرم به‌دست خواهد آمد.

توده بدون چربی: برای محاسبه توده بدون چربی، مقدار چربی به‌دست آمده آزمودنی‌ها (بر حسب کیلوگرم) را از وزن بدن کسر می‌کنیم.

وزن چربی بدن - وزن کل بدن = توده بدون چربی (کیلوگرم)

شاخص توده بدن (Kg/m^2): برای محاسبه این شاخص، وزن بدن آزمودنی‌ها (کیلوگرم) را بر مجذور قد (متر) تقسیم می‌کنیم.

^{۲}(قد) / (وزن) = شاخص توده بدن (BMI)

A) Body composition variables:

Body mass: Subjects will stand on a standard scale (Seca, model 813, made in Germany) with minimal clothes for a few seconds and their body weight will be recorded with an accuracy of 0.1 kg.

Body fat percentage and mass: Estimation of body fat percentage will be done by measuring the subcutaneous fat at three points on the right side of the body (chest, abdomen, and thigh) using a standard caliper (Lafayette, model 01127A, made in the USA). Then, by multiplying the body fat percentage by the subject's body mass, the amount of body fat in kilograms will be calculated.

Fat-free mass: To calculate the fat-free mass, the amount of fat (in kilograms) will be subtracted from the body mass.

Fat-free mass (kg) = body mass (kg) – fat mass (kg)

BMI: To calculate this index, the body mass (in kilograms) will be divided by the square of the body height (in meters).

$$BMI\ (kg/m^2) = body\ mass\ /\ (body\ height)^2$$

ب) متغیرهای جسمانی

قدرت عضلانی بالاتنه: آزمون پرس سینه با استفاده از میله هالتر و وزنه‌های آزاد برای ارزیابی قدرت عضلات بالاتنه آزمودنی‌ها استفاده شده و رکورد آنان (بر حسب کیلوگرم) نسبت به وزن بدن محاسبه خواهد شد.

قدرت عضلانی پایین‌تنه: آزمون اسکوات برای ارزیابی قدرت عضلات پایین‌تنه انجام می‌شود و مقدار رکورد هر فرد نسبت به وزن بدن گزارش خواهد شد.

توان انفجاری: آزمون پرش طول درجا به منظور ارزیابی توان عضلات پایین‌تنه آزمودنی‌ها مورد استفاده قرار می‌گیرد و رکورد آنان بر حسب سانتی‌متر بیان خواهد شد.

سرعت حرکت: از آزمون دوی ۲۰ متر استفاده می‌شود تا توانایی آزمودنی‌ها در عامل سرعت حرکت مورد ارزیابی قرار گیرد. رکورد آزمودنی‌ها با استفاده از کرونومتر و بر حسب ثانیه ثبت خواهد شد.

چابکی: از آزمون رفت و برگشت ۹×۴ متر به‌منظور ارزیابی چابکی آزمودنی‌ها استفاده خواهد شد و رکورد آنان بر حسب ثانیه ثبت می‌شود.

B) Physical fitness factors:

Upper-body muscle strength: The chest press test using barbell bar and free weights will be used to evaluate the upper-body muscle strength (in kilograms) and will be reported in relation to body mass.

Lower-body muscle strength: The squat test will be performed to evaluate the strength of the lower-body muscles, and will be reported in relation to body mass.

Explosive power: The standing long jump test will be used to evaluate the power of the lower-body muscles, and will be expressed in centimeters.

Speed of movement: 20 m sprint test will be used to evaluate the speed of movement using a stopwatch in seconds.

Agility: A 9-m shuttle run test will be used to evaluate the agility of the subjects in seconds.

ج) کنترل انرژی دریافتی

به‌منظور کنترل میزان انرژی دریافتی آزمودنی‌ها پیش از انجام آزمون‌های جسمانی، از آن‌ها خواسته می‌شود تا غذای مصرفی خود را طی ۴۸ ساعت پیش از روز آزمون در برگه‌های استاندارد ثبت رژیم غذایی یادداشت کنند. در خصوص نحوه ثبت غذای مصرفی و واحدهای اندازه‌گیری توضیحات کامل در اختیار تمام آزمودنی‌ها قرار خواهد گرفت. در نهایت، این برگه‌ها تجزیه و تحلیل شده و میزان انرژی دریافتی هر آزمودنی محاسبه می‌شود تا مقادیر آن در تحلیل آماری نتایج حاصل از آزمون‌های جسمانی و ترکیب بدن لحاظ شود.

C) Energy Intake

To control the amount of energy intake before pre-test measurements, participants will be asked to record their food consumption for 48 h prior to the testing day. Complete instructions on how to record food intake and the portion sizes of daily foods will be provided to all subjects.

References

1. Adams K, O’Shea JP, O’Shea KL, Climstein M. The effect of six weeks of squat, plyometric and squat-plyometric training on power production. Journal of applied sport science research. 1992;6(1):36-41.
2. Asadi A, Arazi H. Effects of high-intensity plyometric training on dynamic balance, agility, vertical jump and sprint performance in young male basketball players. Journal of Sport and Health Research. 2012;4(1):35-44.
3. Brzycki M. Strength testing—predicting a one-rep max from reps-to-fatigue. Journal of physical education, recreation & dance. 1993;64(1):88-90.
4. Faul F, Erdfelder E, Lang A-G, Buchner A. G* Power 3: A flexible statistical power analysis program for the social, behavioral, and biomedical sciences. Behavior research methods. 2007;39(2):175-91.
5. Haff GG, Whitley A, McCoy LB, O'Bryant HS, Kilgore JL, Haff EE, et al. Effects of different set configurations on barbell velocity and displacement during a clean pull. Journal of Strength and Conditioning Research. 2003;17(1):95-103.
6. Houtkooper L, Going S. Body composition: how should it be measured? Does it affect sport performance. Sports Science Exchange. 1994;7(5):112-8.
7. Nicol C, Avela J, Komi PV. The stretch-shortening cycle. Sports medicine. 2006;36(11):977-99.

فرم رضایت‌نامه آگاهانه

رضایت‌نامه شرکت در پژوهش با عنوان **مقایسه اثر تمرینات پلایومتریک خوشه‌ای با فواصل استراحتی مختلف بر ترکیب بدن، قدرت و توان عضلانی، سرعت حرکت و چابکی پرسنل نظامی**

**شرکت کننده محترم**<br>بدین وسیله از شما جهت شرکت در پژوهش فوق‌الذکر دعوت به عمل می‌آید. اطلاعات مربوط به این پژوهش در این برگه خدمتتان ارائه شده است و شما برای شرکت یا عدم شرکت در این پژوهش آزاد هستید.<br>شما مجبور به تصمیم‌گیری فوری نیستید و برای تصمیم‌گیری در این باره می‌توانید سوالات خود را از آقایان بهزاد بازگیر و بهزاد طاعتی مقدم بپرسید و با هر فردی که مایل باشید مشورت نمایید. قبل از امضای این رضایت‌نامه مطمئن شوید که متوجه تمامی اطلاعات این فرم شده‌اید و به تمام سوالات شما پاسخ داده شده است.<br>**دکتر بهزاد بازگیر**<br>**مجری پژوهش**

۱- هدف این پژوهش عبارت است از:

- بررسی اثر یک دوره شش هفته‌ای از تمرینات پلایومتریک بر وزن بدن، شاخص توده بدن، درصد چربی بدن، توده بدون چربی، قدرت و توان عضلانی، و سرعت و چابکی پرسنل نظامی

۲- شرکت در این پژوهش کاملاً داوطلبانه است و مجبور به شرکت در این پژوهش نیستید. شرکت در این پژوهش هیچ هزینه‌ای برای شما نداشته و به شما اطمینان داده می‌شود که اگر حاضر به شرکت در این پژوهش نباشید، اختیار تام دارید.

۳- به شما اطمینان داده می‌شود که حتی پس از موافقت با شرکت در پژوهش می‌توانید هر وقت که بخواهید، پس از اطلاع به مجری، از پژوهش خارج شوید و خروج شما از پژوهش باعث پرداخت غرامت نخواهد شد.

۴- نحوه‌ی همکاری شما در این پژوهش به این‌صورت است:

این پژوهش بر روی نیروهای نظامی شهر رشت انجام می‌شود که دارای شرایط زیر باشند:

- محدوده سنی ۲۰ تا ۳۵ سال
- داشتن تمرینات منظم ورزشی به‌منظور برخورداری از سطح آمادگی اولیه جهت شرکت در تمرینات پلایومتریک
- عدم ابتلا به بیماری یا آسیب
- عدم مصرف دخانیات

از بین نیروهای داوطلبی که دارای شرایط فوق باشند، تعداد ۵۲ تا ۶۰ نفر برای شرکت در این پژوهش انتخاب می‌شوند. افراد منتخب به ۴ گروه شامل گروه پلایومتریک سنتی، پلایومتریک ۱۰ (استراحت ۱۰ ثانیه‌ای)، پلایومتریک ۲۰ (استراحت ۲۰ ثانیه‌ای) و پلایومتریک ۳۰ (استراحت ۳۰ ثانیه‌ای) تقسیم می‌شوند. تمرینات پلایومتریک نوعی از تمرینات مقاومتی با استفاده از وزن بدن است که مشخصه اصلی آن اجرای حرکات به‌صورت پرشی یا پرتابی می‌باشد و می‌تواند عملکردهای عضلانی را بهبود بخشد. در گروه پلایومتریک سنتی، تمرینات به صورت تکرارهای پشت سر هم در یک ست اجرا می‌شود؛ در حالی که در سایر گروه‌ها، تعداد تکرارهای هر ست به دو بخش مساوی تقسیم شده و بسته به گروه تمرینی، یک استراحت ۱۰، ۲۰ و یا ۳۰ ثانیه‌ای بین هر بخش از تکرارها در

نظر گرفته خواهد شد. عوامل ترکیب بدن و آمادگی جسمانی، قبل از شروع دوره تمرینات شش هفته‌ای و پس از آن اندازه‌گیری می‌شود.

۵- منافع احتمالی شرکت شما در این پژوهش به این شرح است:
نتایج حاصل از این آزمون‌ها که اطلاعات کاملی از وضعیت ترکیب بدن، آمادگی جمسانی و میزان انرژی دریافتی روزانه شما فراهم می‌کند به رایگان در اختیار شما قرار خواهد گرفت. شما می‌توانید بر اساس این نتایج، برنامه‌ریزی دقیقی برای طراحی تمرینات خود در آینده انجام دهید.

۶- آسیب‌ها و عوارض احتمالی شرکت در این مطالعه به این شرح است:
با توجه به اینکه شما در تمرینات منظم ورزشی شرکت می‌کنید، انجام این آزمون‌ها احتمالا آسیب یا عارضه‌ای برای شما به‌همراه نخواهد داشت. با وجود این، پیش از شروع دوره تمرینات به‌طور کامل با تمرینات پلایومتریک آشنا خواهید شد.

۷- اطمینان داشته باشید که دست اندرکاران این پژوهش، کلیه اطلاعات مربوط به شما را نزد خود به صورت محرمانه نگه‌داشته و فقط اجازه دارند نتایج کلی و گروهی این پژوهش را بدون ذکر نام و مشخصات شما منتشر کنند.

۸- اطلاع داشته باشید که کمیته اخلاق در پژوهش بیمارستان بقیه‌الله با هدف نظارت بر رعایت حقوق شما می‌تواند به اطلاعات شما دسترسی داشته باشد.

۹- آقای **بهزاد بازگیر** به‌عنوان مجری پژوهش جهت پاسخگویی به شما معرفی شده و شما می‌توانید هر وقت مشکلی یا سوالی در رابطه با شرکت در پژوهش مذکور دارید با ایشان در میان گذاشته و راهنمایی بخواهید. شماره تلفن ثابت و همراه ایشان به شرح زیر می‌باشد:

تلفن ثابت: ۰۲۱۸۷۵۵۲۴۰۸ / **تلفن همراه:** ۰۹۳۳۴۷۸۱۱۱۰

۱۰- اگر در حین و بعد از انجام پژوهش هر مشکلی اعم از جسمی و روحی به علت شرکت در این پژوهش برای شما پیش آید، درمان عوارض و هزینه‌های آن و غرامت مربوطه بر عهده مجری پژوهش خواهد بود.

۱۱- این فرم اطلاعات و رضایت آگاهانه در دو نسخه تنظیم شده و پس از امضا یک نسخه در اختیار شما و نسخه دیگر در اختیار مجری پژوهش قرار خواهد گرفت.

اینجانب .... موارد فوق‌الذکر را خواندم و فهمیدم و بر اساس آن رضایت آگاهانه خود را برای شرکت در این پژوهش اعلام می‌کنم.<br>امضای شرکت کننده

اینجانب **بهزاد بازگیر** خود را ملزم به اجرای تعهدات مربوط به مجری در مفاد فوق دانسته و متعهد می‌گردم در تأمین حقوق و ایمنی شرکت کننده در این پژوهش تلاش نمایم.<br>امضای مجری پژوهش

Informed consent form

Informed consent to participate in the research entitled **comparison of cluster sets plyometric training with different rest intervals on body composition, muscular strength and power, speed of movement, and agility in military forces**.

Dear participant
You are hereby invited to participate in the aforementioned research. The information related to this research is presented in this form and you are free to participate or not participate in this research.
You don't have to make an immediate decision, and you can ask your questions to Dr. Behzad Bazgir and Mr. Behzad Taaty Moghadam, and consult with any person you want. Before signing this consent form, make sure that you have understood all the information in this form and that all your questions have been answered.

Dr. Behzad Bazgir
The research conductor

1- The purpose of this research is:

- Investigating the effect of a six-week period of plyometric training on body mass, body mass index, body fat percentage, fat-free mass, muscular strength and power, speed of movement and agility in military forces.

2- Participation in this research is completely voluntary and you are not forced to participate in this research. There is no cost to you to participate in this research, and you are assured that if you are not willing to participate in this research, you have full authority.

3- You are assured that even after agreeing to participate in the research, you can withdraw from the research whenever you want, after informing the conductor, and your withdrawal from the research will not result in payment of compensation.

4- The way of your cooperation in this research is as follows:

This research is conducted on the military forces of Rasht city who have the following conditions:

- Age range of 20 to 35 years
- Having regular exercise training in order to have a basic level of physical fitness to participate in the plyometric training protocol
- No illness or injury
- No smoking

Among the volunteers who have the above conditions, 52 to 60 persons will be selected to participate in this research. The selected subjects will be divided into 4 groups including traditional plyometric group, plyometric 10 (10 second rest), plyometric 20 (20 second rest) and plyometric 30 (30 second rest).

Plyometric exercises are a type of resistance exercises using body weight, the main characteristic of which is the execution of tasks in the form of jumping or throwing, and it can improve muscle performance. In

the traditional plyometric group, the exercises will be performed as continuous repetitions in each set; while in other groups, the number of repetitions in each set will be divided into two equal clusters and depending on the training group, a 10, 20, or 30 s rest will be considered between the clusters. Body composition and physical fitness factors will be measured before and after the six-week training period.

5- The possible benefits of your participations in this research are as follows:

- The results of these tests, which provide complete information about your body composition, physical fitness, and your daily energy intake, will be provided to you for free. Based on these results, you can make more detailed plan for designing your exercises in the future.

6- Possible damages and complications of participating in this study are as follows:

- Considering that you participate in regular exercise training, performing these tests will probably not cause any damage or complications for you. However, you will be thoroughly familiar with plyometric exercises before starting the training period.

7- Make sure that the persons involved in the research procedures keep all the information related to you confidential and they are only allowed to publish the general and group results of this research without mentioning your name and details.

8- Be aware that the Research Ethics Committee of Baqiyatallah Hospital can access your information for the purpose of monitoring the observance of your rights.

9- Dr. Behzad Bazgir has been introduced to you as the research conductor to answer your questions and you can contact with him and ask for guidance whenever you have any concerns related to participating in this research. His contact information is as follows: Phone: 02187552408 / Mobile: 09334781110

10- If during and after the research, any problem, whether physical or mental, occurs to you due to participating in this research, the treatment of complications, costs and the related compensation will be the responsibility of the research conductor.

11- This informed consent form will be prepared in two copies and after signing, one copy will be delivered to you.

I, …………..., have read and understood the above-mentioned information, and based on that, I declare my informed consent to participate in this research.<br><br>Signature of the participant

I, Behzad Bazegir, consider myself bound to fulfill the obligations related to the executive in the above provisions, and I undertake to ensure the rights and safety of the participants in this research.<br><br>Signature of the research conductor
